# Authors' Response to Zimmer et al

**DOI:** 10.1371/journal.pmed.0030365

**Published:** 2006-08-29

**Authors:** Roberto Stasi, Sergio Amadori, John Osborn, Adrian Newland, Drew Provan

**Affiliations:** Ospedale Regina Apostolorum, Rome, Italy; University “Tor Vergata” Rome, Italy; University “La Sapienza” Rome, Italy; Barts and the London School of Medicine and Dentistry London, United Kingdom

We wish to thank Dr. Zimmer and colleagues [1] for critically reviewing our paper [2] and bringing to our knowledge their results. We partly agree with their comments, especially the part when they affirm that a direct comparison of the results is difficult.

First of all, the design of the two investigations was different: ours was prospective and theirs was retrospective. Secondly, their data can hardly be interpreted and so cannot be a matter of contention. In fact, they merely report a mean platelet count of 88 × 10^9^/l for the untreated group of 62 idiopathic thrombocytopenic purpura patients and of 66 × 10^9^/l for the 31 patients later reclassified as having chronic idiopathic thrombocytopenic purpura. More importantly, they do not specify the number of their patients with a platelet count between 100 × 10^9^/l and 150 × 10^9^/l, i.e., the class of individuals that was the focus of our study.

As an additional confounding factor, they report a follow-up period of 1.9 to 59 months for the entire untreated group. If a median is not reported, this does not make much sense statistically. Theoretically, 31 patients might have been followed for 1.9 months, 30 patients for six months, and one single patient for 59 months. If this was the case, no wonder they did not observe a single case of autoimmune disease in their cohort. We do not share Dr. Zimmer's point about a platelet count of 51 × 10^9^/l to 100 × 10^9^/l as equivalent to a higher count. Subjects who have a platelet count in the range of 50 × 10^9^/l to 80 × 10^9^/l are limited in their performance of particular physical jobs or traumatic activities such as contact sports. Besides, current guidelines suggest that a “safe” platelet count for major surgery, cesarean section, and spinal or epidural anesthesia should be at least 80 × 10^9^/l [3]. Therefore, these patients may occasionally require an evaluation and possibly treatment that is not required for those with a borderline thrombocytopenia.

Finally, we definitely rebut the issue of creating an unneeded clinical entity. The goal of our study was simply to describe the long-term outcome of individuals who were incidentally found with a platelet count between 101 × 10^9^/l and 150 × 10^9^/l. The terms “borderline thrombocytopenia” should be interpreted only as the definition of a count in that range, not as a new clinical entity. In fact, we have clearly underlined that the majority of individuals will retain their borderline platelet count indefinitely without developing diseases. Only a prospective case-control study would establish whether such individuals have a higher risk of developing autoimmune disorders than the general population. Until then, these cases should be interpreted only as healthy individuals with a platelet count in the lower range of normal.
